# High-dose liraglutide improves metabolic syndrome in poor responders to bariatric surgery

**DOI:** 10.3389/fnut.2023.1183899

**Published:** 2023-09-13

**Authors:** Federica Vinciguerra, Luigi Piazza, Carla Di Stefano, Claudia Degano, Alfredo Pulvirenti, Roberto Baratta, Lucia Frittitta

**Affiliations:** ^1^Department of Clinical and Experimental Medicine, University of Catania, Catania, Italy; ^2^General and Emergency Surgery Department, Garibaldi Hospital, Catania, Italy; ^3^Bioinformatics Unit, Department of Clinical and Experimental Medicine, University of Catania, Catania, Italy; ^4^Endocrinology Unit, Garibaldi Hospital, Catania, Italy; ^5^Diabetes and Obesity Center, Garibaldi Hospital, Catania, Italy

**Keywords:** obesity, bariatric surgery, metabolic syndrome, liraglutide, weight loss

## Abstract

**Background:**

Bariatric surgery (BS) represents the most effective therapy for obesity class III, or class II with at least one weight-related comorbidity. However, some patients have insufficient weight loss or clinically relevant weight regain after a successful primary procedure. This study aimed to assess the efficacy of liraglutide treatment on weight loss, body composition and improvement of metabolic syndrome (MS) in patients defined as poor responders after BS.

**Methods:**

The study involved 59 non-diabetic adults with obesity (M/F: 17/42, age: 38.6 ± 11.8 years, BMI 38.3 ± 5.5 kg/m^2^) who had been treated with BS and experienced a poor response, categorized as either IWL (insufficient weight loss) or WR (weight regain). All patients were prescribed pharmacological therapy with liraglutide and attended nutritional counseling. Anthropometric and clinical measurements, body composition and the presence of MS defined according to the ATP-III classification were evaluated before starting liraglutide and after 24 weeks of treatment.

**Results:**

After 24 weeks of treatment with liraglutide, the mean weight loss was 8.4% ± 3.6% with no difference between gender, bariatric procedure, or type of poor response (IWL or WR). A significant decrease in fat mass, free-fat mass and total body water was documented. After 24 weeks, patients presented significantly lowered fasting glucose, total cholesterol, triglycerides, AST and ALT. The prevalence of MS was reduced from 35% at baseline to 1.6% after 24 weeks. No patients discontinued the treatment during the study.

**Conclusion:**

In patients who experience poor response after BS, liraglutide is well tolerated and promotes significant weight loss, ameliorates cardiometabolic comorbidities, and reduces the prevalence of MS.

## 1. Introduction

Obesity is a complex chronic relapsing disease, resulting from the interaction between multiple environmental, social, genetic and epigenetic causes, and from changes in neuroendocrine mechanisms regulating energy balance and body weight (1). In 2020, it was estimated that 764 million adults globally or 15% of the world’s population are living with obesity (2). Bariatric surgery represents the most effective therapy for obesity class III, or class II with at least one weight-related comorbidity, resulting in significant weight loss and improving metabolic and cardiovascular comorbidities, quality of life, and life expectancy (3, 4). However, for partially unknown reasons, a poor response to bariatric surgery is evident in a subgroup of patients that have insufficient weight loss (IWL), generally defined as an initial loss of <50% of excess weight loss (EWL), or clinically relevant weight regain (WR) after a successful primary procedure (5). More than 1 out of 3 patients with previous gastric bypass (RYGB) experience weight recidivism, defined as regain of ≥25% of total weight loss (6). This long-term issue of bariatric surgery may affect the general benefits of weight loss, favoring the persistence or re-onset of obesity-related comorbidities with a consequent worsening of the quality of life and a profound frustration of failure for the patient (7–10). Management for non-responders to bariatric surgery includes lifestyle interventions and psychological counseling. In addition, antiobesity medications (AOMs) may provide a valuable adjunct to lifestyle interventions, which typically have a limited effect on weight loss, to help people achieve and maintain healthy behaviors consistent with sustainable weight loss. European guidelines recommend the use of AOMs in adult patients with BMI ≥30 kg/m^2^ or having a BMI of ≥27 kg/m^2^ and at least one weight-related comorbidity (11, 12). Liraglutide 3.0 mg/day, a weight management drug approved by the European Medicines Agency, is an acylated analog of human GLP-1, that is a physiological regulator of appetite. Liraglutide 3.0 mg/day induces weight loss in subjects with overweight and obesity by reducing appetite and caloric intake, without increasing energy expenditure (13, 14). High-dose liraglutide has been shown to significantly enhance and sustain weight loss in patients who have previously lost weight on a low-calorie diet (15). However, to date, studies on the efficacy of liraglutide in patients with poor response after bariatric surgery are few and data on body composition changes and metabolic syndrome are lacking. This study aimed to assess the efficacy of liraglutide treatment on weight loss, body composition and improvement of metabolic syndrome in patients defined as poor responders after bariatric surgery.

## 2. Materials and methods

### 2.1. Study design and population

This study was conducted at the Diabetes and Obesity Center in Garibaldi Hospital, Catania, Italy among 59 non-diabetic adult patients with obesity who had been treated with bariatric surgery (38 sleeve gastrectomy and 21 one-anastomosis gastric bypass) and experienced a poor response. Poor response after bariatric surgery was categorized as either IWL or WR. IWL was defined as an initial weight loss of less than 50% of excess weight loss (EWL), WR was defined as a gain of at least 15% of the weight lost after bariatric surgery (16). WR was calculated using the following formula: (current weight − nadir weight)/(pre-bariatric weight − nadir weight) × 100 (17).

In our Medical Center, patients who undergo a bariatric surgery procedure are followed by a multidisciplinary team of bariatric physicians, dietitians, psychologists and surgeons for preoperative assessment of physical, metabolic, and psychologic status and life-long management of weight loss, comorbidities and prevention of complications. Demographic, anthropometric, and clinical data were retrospectively collected from the patient’s electronic records.

The study sample was represented by patients aged 18–65 years who started liraglutide as pharmacological therapy for the management of WR or IWL after bariatric surgery from January 2016 to July 2022. The exclusion criteria were current use of other weight loss medication, pregnancy or breastfeeding, presence of acute and/or severe organ disease, Type 1 or 2 diabetes, congestive heart failure (NYHA class IV), personal or family history of medullary thyroid carcinoma (MTC) or multiple endocrine neoplasia type 2 (MEN2), any personal history of non-familial MTC, and personal history of acute or chronic pancreatitis.

The Ethical Committee Catania 2 approved the study (94/CECT2, 27/09/2022). Investigations were performed in accordance with the principles of the Declaration of Helsinki.

During the examined period, 103 patients with WR or IWL had been referred to our center. All patients were evaluated for a surgical cause of poor response to bariatric surgery. The clinical decision to proceed with nutritional intervention, pharmacotherapy or revisional bariatric surgery has been based on a complete multidisciplinary assessment of the patient, taking also into account the patient’s risk profile and preferences. Fifty-nine patients having been prescribed pharmacological therapy with liraglutide met inclusion criteria. The final sample was composed of 14 patients who experienced IWL (EWL% 37.14 ± 7.6) and 45 patients who experienced WR (38.6% ± 20.4%) after 20 ± 6 months from the bariatric procedure (sleeve gastrectomy or one-anastomosis gastric bypass). Liraglutide treatment was started at a dose of 0.6 mg once daily and was increased by 0.6 mg weekly to reach the minimum effective dose (mean dosage 2.4 mg). In Italy, pharmacological therapy for obesity is not reimbursed and the expensive cost represents one of the main obstacles to treatment because not all patients are able to purchase it.

All patients attended nutritional counseling with a registered dietitian who recommended a post-bariatric nutritional regimen with 1.5 g/kg ideal body weight protein intake, low glycaemic index, and high fiber–containing foods (18), emphasizing changing dysfunctional eating habits. Moderate-intensity physical activity for 150 min/per week was also recommended.

Side effects, including gastrointestinal tolerance, were assessed at each visit.

### 2.2. Anthropometric and clinical measurements and body composition

All the patients underwent a physical examination at baseline and after 24 weeks with the assessment of height, body weight (BW) and waist circumference (WC). Blood pressure was measured twice with the subject in a sitting position after a minimum of 5 min of acclimatization and before blood sampling. The mean of the two blood pressure measurements was used in the analysis.

### 2.3. Body composition

Body composition was assessed after 3 h fast using bioelectrical impedance analysis (BIA; TANITA MC 180 MA measuring station). Fat mass (FM), fat-free mass percentage (FFM), and total body water (TBW) were evaluated.

### 2.4. Blood analysis

Blood specimens were obtained after an overnight fast and glucose, total cholesterol, high-density lipoprotein (HDL) cholesterol, triglycerides (TG), alanine transaminase (AST), and aspartate transaminase (ALT) were measured using commercially available kits. All laboratory analyses were performed in a single clinical laboratory according to standard procedures. Low-density lipoprotein (LDL) cholesterol was calculated using the Friedwald formula (total cholesterol – HDL) – (TG/5).

### 2.5. Metabolic syndrome

The presence of metabolic syndrome was evaluated before starting liraglutide and after 24 weeks of treatment. Metabolic syndrome was defined according to the ATP-III classification (19), which requires three or more of the following five components: large waist circumference (>102 cm in men and >88 cm in women), hypertriglyceridemia (>150 mg/dL) or antihyperlipidemic treatment, low HDL cholesterol level (<50 mg/dL in men and <40 mg/dL in women), elevated blood pressure (systolic > 130 mmHg and/or diastolic > 85 mmHg and/or antihypertensive treatment), and elevated fasting plasma glucose (>100 mg/dL).

### 2.6. Statistical analysis

Continuous variables are described as mean ± SD, while categorical variables are expressed as count and percentage. All continuous variables were normally distributed (Kolmogorov–Smirnov test). Longitudinal changes were evaluated by paired Student’s t-test. For categorical variables, statistical significance between groups was established using Chi-Square test. Changes over time in BMI values were evaluated by ANOVA followed by a post-hoc analysis with Fisher’s protected least-significant difference test (PLSD). The significance limit was set at *p*-values of <0.05. The data analyses were performed using the JMP statistical package software (version 10.0).

The sample size was calculated considering an expected difference of 10 kg in body weight between the beginning and after 24 weeks of treatment, with a SD of 18 kg, since the study includes subjects with previous bariatric surgery of any type. Accordingly, it was established that at least 46 subjects were needed to achieve a sample of 27 subjects with an alpha error of 0.05% and a power equal to 80%, considering a drop out in treatment with liraglutide equal to 40%. A drop-out of 40% was estimated considering the study’s retrospective design, the lack of information relative to the 6 months follow-up for some eligible patients, the cost of the pharmacotherapy and the tolerability to gastrointestinal adverse events.

## 3. Results

### 3.1. Anthropometric and clinical outcomes

The entire population included 59 subjects, 71% of whom were female (M/F = 17/42). The mean age was 38.6 ± 11.8 years, and females were significantly older than males (41.7 ± 11.5 vs. 30.3 ± 0.1, *p* = 0.006). The mean BMI was 38.3 ± 5.5 kg/m^2^ with no difference between genders.

No difference in age, gender, body weight and BMI was found between patients with IWL or WR.

Clinical characteristics at baseline and after treatment are represented in Table 1.

**Table 1:** Clinical and biochemical parameters at baseline and after 24 weeks of treatment with liraglutide.

Clinical and metabolic parameters	Baseline	After 24 weeks	value of *p*
Body weight (kg)	101.8 ± 17.9	93.3 ± 17.6	<0.0001
BMI (kg/m^2^)	38.2 ± 5.7	35.1 ± 5.6	<0.0001
WC (cm)	123.2 ± 13.3	113.9 ± 13.5	<0.0001
SBP (mmHg)	125.9 ± 10.8	119.2 ± 7.6	<0.0001
DBP (mmHg)	80.5 ± 7.6	75.6 ± 5.2	<0.0001
Glucose (mg/dL)	99.3 ± 12.4	85.8 ± 7.5	<0.0001
Total cholesterol (mg/dL)	186.1 ± 29.2	176.3 ± 29.4	0.009
HDL Cholesterol (mg/dL)	50.7 ± 12.6	51.3 ± 13.5	0.71
Triglycerides (mg/dL)	112.3 ± 47.3	82.5 ± 33.2	<0.0001
LDL Cholesterol (mg/dL)	112.9 ± 30.2	108.6 ± 30.3	0.2
AST (mg/dL)	21.9 ± 8.4	18.1 ± 5.4	<0.0001
ALT (mg/dL)	27.9 ± 15.0	17.2 ± 7.3	<0.0001

Data are shown as mean ± SD. BMI, body mass index; WC, waist circumference; SBP, systolic blood pressure; DBP, diastolic blood pressure; LDL, low-density lipoprotein; HDL, high-density lipoprotein; AST, alanine transaminase; ALT, aspartate transaminase.

After 24 weeks of treatment with liraglutide, the mean percentage weight loss in the entire cohort was 8.4% ± 3.6% with no difference between genders, bariatric procedure, or type of poor response (Table 2).

**Table 2:** Percentage of weight loss (WL) according to gender, bariatric procedure, or cause of poor response.

	WL (%)		Value of *p*
**Gender**			
*Females* *Males*	8.6±3.8 7.8±3.1	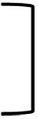	0.43
**Surgical procedure**			
*SG* *OAGB*	8.8±3.5 7.8±3.8	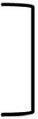	0.31
**Poor-response**			
*IWL* *WR*	7.6±2.5 8.6±4	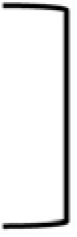	0.35

SG, sleeve gastrectomy; OAGB, one anastomosis gastric bypass; IWL, insufficient weight loss; WR, weight regain.

**Figure 1:**
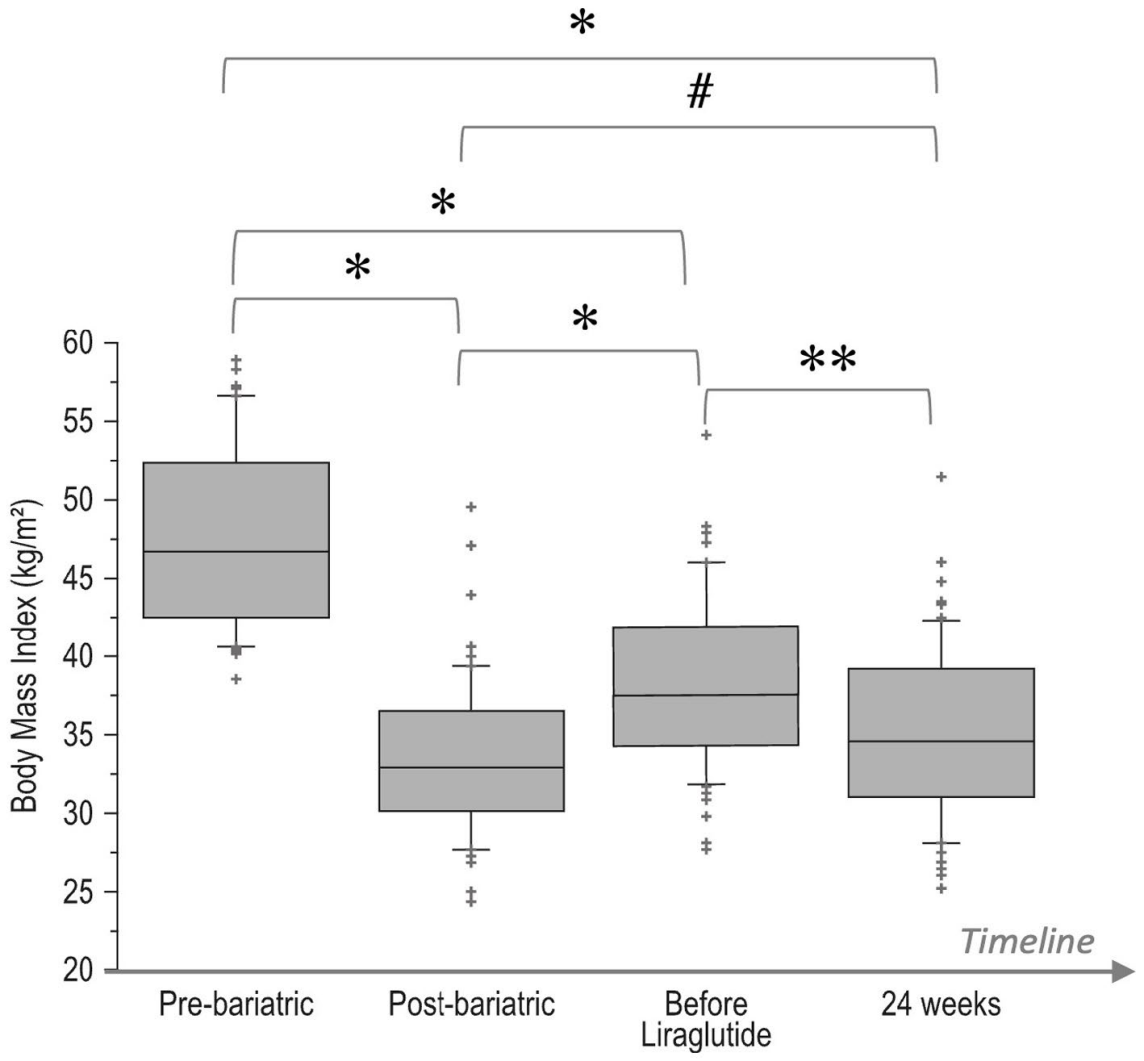
Boxplot of changes over time of BMI values: pre-bariatric, at nadir post-bariatric, at the start of liraglutide, and after 24 weeks of treatment. ^*^p < 0.0001, ^**^p = 0.0002, ^#^*p* = 0.09. Bottom and top of box represent the second and third quartiles, respectively. The ends of the whiskers represent the first and fourth quartiles.

**Figure 2:**
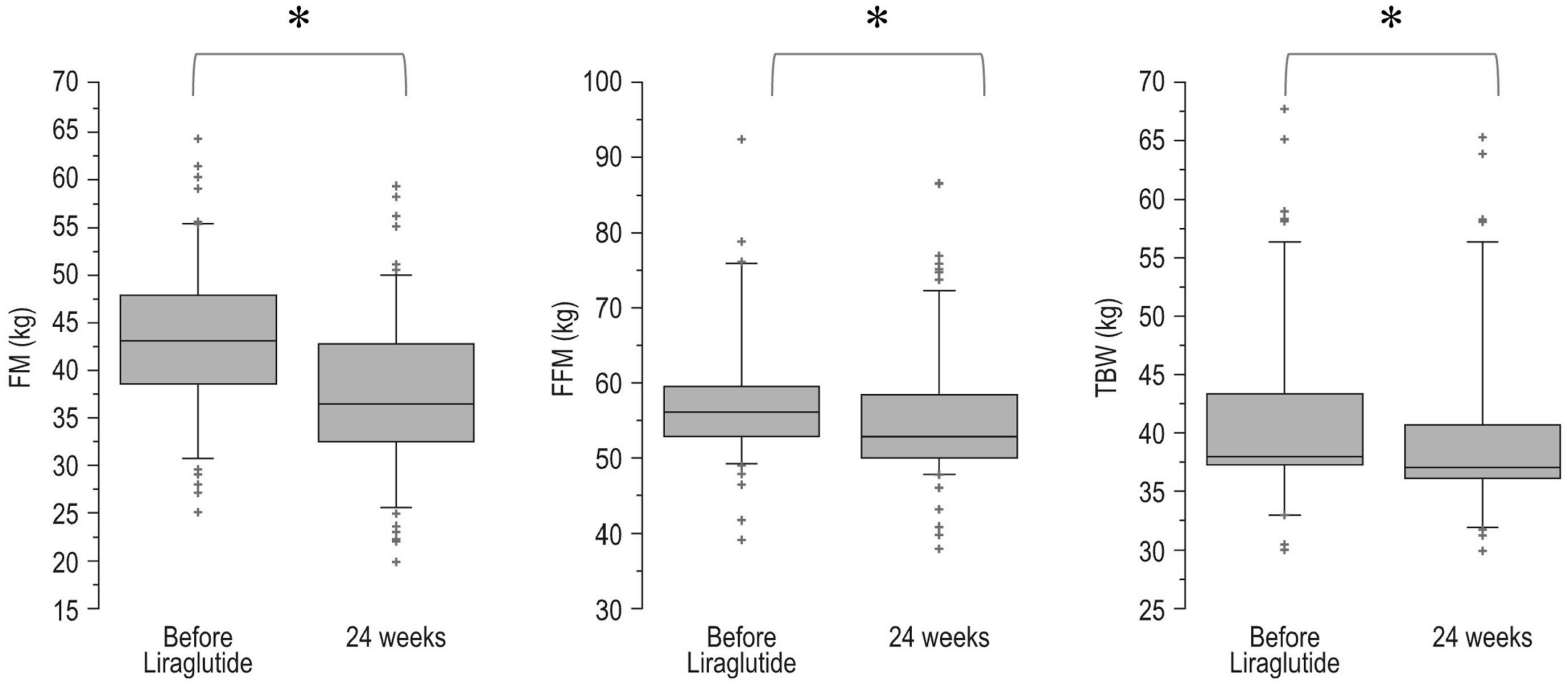
Boxplot of body composition before and after 24 weeks of treatment with liraglutide. FM, fat mass; FFM, free-fat mass; TBW, total body water. ^*^p < 0.0001. Bottom and top of box represent the second and third quartiles, respectively. The ends of the whiskers represent the first and fourth quartiles.

Changes over time of BMI values (pre-bariatric, at nadir post-bariatric, at the start of liraglutide, and after 24 weeks of treatment) are illustrated in Figure 1. In detail, after the bariatric procedure BMI decreased significantly from baseline values (47.7 ± 5.8 kg/m^2^), reaching a nadir of 33.4 ± 4.9 kg/m^2^ (*p* < 0.0001). Then, a significant WR occurred (BMI 38.3 ± 5.6 kg/m^2^, *p* < 0.0001 vs. nadir). After liraglutide treatment, a significant reduction in BMI (35.1 ± 5.6 kg/m^2^, *p* = 0.0002) was observed with values that were not different from those of the post-bariatric nadir (*p* = 0.09). Body composition analysis showed a significant (<0.0001) decrease in FM (−5.6 ± 2.5 kg), FFM (−2.8 ± 1.8 kg) and total body water (−1.5 ± 0.8 kg; Figure 2).

Most patients (*n* = 52, 88.2%) lost at least 5% of their body weight; in detail, 11.8% (*n* = 7) lost between 0 and 5%, 59.3% (*n* = 35) between 5 and 10%, 20.3% (*n* = 12) between 10 and 15% and 8.5% (*n* = 5) more than 15% of their body weight.

At 24 weeks, patients displayed a significant reduction in systolic and diastolic blood pressure (Table 1).

### 3.2. Biochemical outcomes

After 24 weeks, patients presented significantly ameliorated metabolic and lipid profiles with lowered fasting glucose, total cholesterol, triglycerides, AST and ALT (Table 1).

### 3.3. Metabolic syndrome

At baseline, 35% (*n* = 21) of patients fulfilled the criteria for MS. After 24 weeks, MS was still present in only one patient (1.6%). In addition, at baseline, 100% had a high WC, 39% (*n* = 23) had hypertension, 61% (*n* = 36) had impaired fasting glucose, 24% (*n* = 14) had low HDL-cholesterol and 13% (*n* = 8) had hypertriglyceridemia.

After 24 weeks, high WC was present in 93% (*n* = 55), hypertension was still present in only 11% (*n* = 10) of patients, low HDL-cholesterol in 15% (*n* = 9), hypertriglyceridemia in 5% (*n* = 3, *p* = 0.006 vs. baseline) and all patients obtained normalization of glycemic status.

### 3.4. Adverse events

No serious adverse events were recorded. No patients discontinued the treatment during the 24 weeks. The most frequent adverse events were mild nausea at the beginning of the treatment and constipation.

## 4. Discussion

Our real-life study shows that liraglutide is an effective treatment for inducing weight loss and improving metabolic syndrome in patients with poor response to bariatric surgery. After 24 weeks of treatment, a significant body weight loss, higher than 8%, was reached with a predominant reduction of fat mass and amelioration of cardio-metabolic parameters.

To our knowledge, this is the first study assessing the efficacy of liraglutide not only on weight loss and body composition but also on the improvement of MS in non-diabetic patients with IWL or WR.

The management of poor response after bariatric surgery represents a challenge because the treatment options for patients with IWL or WR following bariatric surgery are limited. Reinforcing nutritional, physical, and psychological consultations is the first therapeutical approach. Revision bariatric procedures have higher rates of complications than the primary intervention. Recently, we have demonstrated the short-term effectiveness of a nutritional approach based on ketogenic diets in patients with IWL and WR (20). However, no data on long-term efficacy are available. Furthermore, it is a very strict regimen that requires rigorous compliance. Pharmacotherapy is recommended by the European Association for the Study of Obesity (EASO) but is still underutilized. Available data suggest that weight loss medications could offer a significant adjunctive benefit to lifestyle and behavioral modifications in the management of poor responders to bariatric surgery (21).

Our findings are consistent with existing retrospective (22–25) and prospective (26) evidence on the use of weight loss medications in non-diabetic patients with previous bariatric surgery.

In our cohort, the prevalence of responders (weight loss > 5%) was higher than those observed in SCALE obesity trial (27); more than 88% of our bariatric patients lost more than 5% of baseline body weight with respect to 60% of non-surgical patients with obesity. The different weight loss responses might be partially explained by differences in diet counseling provided. The nutritional recommendations for post-bariatric patients are adapted for limited gastric capacity and nutritional malabsorption which generally reduce food and calorie intake.

Liraglutide’s effect in attenuating the sense of hunger in post-bariatric poor responder patients seems to improve adherence to post-bariatric nutritional recommendations and might restore the effectiveness of the bariatric procedure resulting in a greater amount of total weight loss than in non-bariatric patients with obesity.

In contrast to recent retrospective data comparing pharmacotherapy (including phentermine, topiramate, liraglutide, and orlistat) and revision surgery in poor responders after bariatric surgery (28), our study did not find any difference in weight loss outcomes between patients with IWL and WR. Additionally, in the Dharmaratnam study, the most common medication used was phentermine and, unlike our results, the overall weight loss achieved in their cohort was modest (1.9% ± 4.3% for WR and 0.7% ± 4.2% for IWL) (28).

Moreover, no difference in weight loss regardless of the bariatric procedure was found in our patient cohort. These results are similar to those reported from retrospective studies of Muratori et al. (25) and Wharton et al. (24).

Weight loss was accompanied by improvements in cardiometabolic risk factors including reductions in waist circumference, blood pressure, glucose, and lipid levels. These results do not agree with recent data evaluating the effectiveness of liraglutide in managing IWL and WR after primary vs. revisional bariatric surgery which demonstrated no improvement in cardiometabolic outcomes for primary patients, despite a significant weight loss (29). Elhag et al. explained these results by the fact that cardiometabolic parameters of their patients were already normal before starting liraglutide.

Poor responses to bariatric surgery compromise the benefits of weight loss, promoting the persistence or recurrence of obesity-related comorbidities.

Our study demonstrated a significant reduction of main obesity-related comorbidities such as hypertension and dyslipidemia in most of the patients and even the normalization of glucose metabolism in the overall population.

Based on the amelioration of cardiometabolic parameters, the prevalence of MS in our cohort decreased significantly.

After 24 weeks of treatment, liraglutide induced a significant improvement in liver parameters in our patient cohort. A possible beneficial hepatic effect has been demonstrated with the use of GLP1 receptor agonists (GLP1-RA) and liraglutide has been the most studied in nonalcoholic fatty liver disease (NAFLD).

Recent evidence has shown that it also improves the hepatic histological components of NAFLD and reduces the progression of fibrosis (30).

Gastrointestinal adverse events were mild and did not lead to treatment discontinuation. This represents a critical issue in post-bariatric patients who frequently experience gastrointestinal symptoms related to anatomical and physiological modifications following the surgery (31). Our results suggest that liraglutide used in bariatric patients has no side effects other than those reported in non-surgical patients with obesity.

Limitations of our study include a retrospective analysis, a relatively short follow-up, and a small sample size.

Poor response to bariatric surgery represents a challenge in the management of obesity, especially with the increase in the number of patients receiving surgical therapy. It can be considered a long-term complication of bariatric surgery because it promotes the persistence or recurrence of obesity-related comorbidities and consequently worsens the patient’s quality of life causing a deep sense of failure and frustration. It confirms that obesity is a serious, complex, relapsing chronic disease process that needs a long-life, multimodal and sequential approach. A safe drug therapy, effective in the control of satiety, inducing weight loss and also ameliorating cardiometabolic outcomes represents a valuable option for the treatment of poor response to bariatric surgery.

## 5. Conclusion

In patients who experience IWL or WR after bariatric surgery, liraglutide is well tolerated and promotes significant weight loss, ameliorates cardiometabolic comorbidities, and reduces the prevalence of metabolic syndrome, representing a useful tool for the management of poor response to bariatric surgery.

## Data availability statement

The raw data supporting the conclusions of this article will be made available by the authors, without undue reservation.

## Ethics statement

The studies involving humans were approved by Ethics Committee of Garibaldi Hospital (94/CECT2, 27/09/2022), Catania. The studies were conducted in accordance with the local legislation and institutional requirements. The participants provided their written informed consent to participate in this study.

## Author contributions

FV, LP, CS, CD, AP, RB, and LF contributed to the study conception and design. All authors contributed to the article and approved the submitted version.

## Funding

The editorial assistance was provided by Airon Communication through a Novo Nordisk S.p.A. unconditional grant. Novo Nordisk S.p.A. had no role in the study design, conduct of the study, collection, management, analysis and interpretation of the data; or the preparation and review of the manuscript.

## Conflict of interest

The authors declare that the research was conducted in the absence of any commercial or financial relationships that could be construed as a potential conflict of interest.

## Publisher’s note

All claims expressed in this article are solely those of the authors and do not necessarily represent those of their affiliated organizations, or those of the publisher, the editors and the reviewers. Any product that may be evaluated in this article, or claim that may be made by its manufacturer, is not guaranteed or endorsed by the publisher.
